# Supplementary material for: Unsupervised approach to decomposing neural tuning variability
Source: Nat Commun. 2023 Apr 21;14:2298. doi: 10.1038/s41467-023-37982-z (PMC10121715; doi:10.1038/s41467-023-37982-z)
Supplement: Supplementary file 1 — Supplementary Information [file 41467_2023_37982_MOESM1_ESM.pdf]

Supplementary information for

Unsupervised approach to decomposing neural tuning variability

Rong J.B. Zhu[1,2] and Xue-Xin Wei[3,4,5,6]

1) Institute of Science and Technology for Brain-Inspired Intelligence, Fudan University
2) MOE Key Laboratory of Computational Neuroscience and Brain-Inspired Intelligence, and MOE Frontiers Center for Brain Science, Shanghai, China
3) Department of Neuroscience, The University of Texas at Austin, Austin
4) Department of Psychology, The University of Texas at Austin
5) Center for Perceptual Systems, The University of Texas at Austin
6) Center for Theoretical and Computational Neuroscience, The University of Texas at Austin

Correspondence: rongzhu@fudan.edu.cn; weixx@utexas.edu

Supplementary Note 1: Quantitative evaluation

We performed analysis to quantify the fitting performance of Pf-PCA in comparison to the Modulated Poisson model [25] both in simulated data and V1 dataset.

Because true Poisson mean $\lambda_i(s)$ is known in simulated data, we can directly use the mean squared prediction error to check our Poisson fPCA model. We defined MSPE $= \sum_i \sum_s (\hat{\lambda}_i(s) - \lambda_i(s))^2$, where, for our Poisson fPCA model, $\hat{\lambda}_i(s) = \exp(\hat{f} + \hat{\alpha}_1 \hat{\phi})$ is the fitted mean at stimulus s. For the modulated Poisson model, the fitted mean is its MAP estimate. We used the ground truth of four types of fluctuations with the bell-shaped tuning curves (Fig. 2) as the Poisson mean, then generated 20 datasets for each type from the Poisson distribution. Given these simulated datasets, we calculated the prediction error in the firing rate as shown above. We summarized the ratio of the prediction error of our Poisson fPCA model to that of the modulated Poisson model in the form of a histogram (Supplementary Fig. 1).

For the V1 datasets, true Poisson mean $\lambda_i(s)$ is unknown. For the purpose of model comparison, we performed a K-fold cross-validation, where K is the length of stimuli. For performing the cross-validation, we randomly chose a stimulus trial per block as the hold-out data, and used the remaining trials in that block as the training data. Then we fitted both our model and the modulated Poisson model to the training data and compared the mean squared prediction error of both models for the hold-out data. The ratio of the cross-validated prediction error of Poisson fPCA to that of modulated Poisson was reported in Supplementary Fig. 2. To further evaluate the models, we also compared the conditional probability given the predicted $\hat{\lambda}_i(s)$ of the hold-out data. The ratio of the cross-validated likelihood of Poisson fPCA to that of modulated Poisson was reported in Supplementary Fig. 2.

Supplementary Note 2: Recovering mixed fluctuations

Fig. 2 has shown that the tuning fluctuations could be captured by a single functional component, however, fluctuations in real data may consist of a mixture of different kinds of variability, e.g., lateral shift and the multiplicative gain. Therefore we next examine if our method would work given such a more complex structure. We mixed two of the single kinds above (see Supplementary Fig. 3) and stimulated the count matrices from the neural tuning. Supplementary Fig. 3 shows that the recovered components from Pf-PCA are close to the ground-truth fluctuation, which suggests that the method can also successfully identify latent structures in more complex cases.

Supplementary Note 3: Recovering fluctuations for monotonic tuning curves

The response functions of neurons can often be monotonic, e.g., time course in the LIP area [74], response to the motion strength in macaque MT area [104] and time course in the Olfactory bulb [105]. In this section, we address this class of tuning curve shape by using sigmoid functions as a representative example. The fluctuations considered here are the multiplicative gain, the tuning shift, and the slope change (leftmost panel of Supplementary Fig. 5). Similar to the results reported for bell-shape tuning curves, we found that Pf-PCA could recover the basic structure of fluctuations well (see Supplementary Fig. 5). It also outperforms the alternative methods (regular PCA and μ-PCA).

Supplementary Note 4: The impact of sample size

The results reported in Fig. 3 were obtained based on hundreds of blocks of data (400 for the D1-D3, 200 for the D5-D7). To examine the impact of sample size, we ran Pf-PCA on subsets of V1 data by taking 25, 50, or 100 blocks of each dataset. These results were shown in Supplementary Fig.7. Comparing to the results in Fig.3, we found that the variance explained by the first fPC and the significance of regression analysis is robust with respect to the sample size. However, reducing sample size has an impact on the “fraction" index when regressing the first fPC against the recovered mean component.

Supplementary Note 5: Further analysis on the FI and 8-way classification task

While our results are consistent with [30] in that both studies found that increased population activities do not lead to a substantial increase in population FI, we also noticed some subtle discrepancies between the two studies, because [30] suggested that there was a minimal change of population FI when population activity changes. We believe that the difference lies in the difference in the analysis methods. Our results were based on direct estimation of FI, while [30] used 8-way classification by splitting the data into groups with high/low population activity. FI by definition is a local measure of discriminability, thus 8-way classification may not represent an accurate measure of FI. Additionally, by splitting the data into two groups [30], there are likely still a substantial amount of tuning fluctuations within each group. Such uncertainty might dominate the classification performance, reducing the amount of change when comparing the high/low groups.

To further understand the difference between the two methods, we performed analysis using synthetic data with known ground truth (Fig. 6d). We first generated simulated data which match the summary statistics of the real data, and applied our analysis pipeline to estimate the FI. Next, in a separate analysis, we performed 8-way classifications using the procedure in [30] on the synthetic data. We found that i) our method could reliably estimate the FI, both at the level of individual neurons (Supplementary Fig. 14) and the neural populations (Fig. 6d); ii) performance based on classification analysis could not be mapped onto the change of FI in obvious ways - higher FI (with decreased population firing rate) does not necessarily leads to better classification performance (Supplementary Fig. 15). We also verified our classification procedure on the datasets used in [30], and we were indeed able to replicate the classification results in [30] (Supplementary Fig. 15). These analyses suggest that the two analysis methods may capture different aspects of the code, and generally speaking, 8-way classification task does not lead to accurate characterization of the FI.

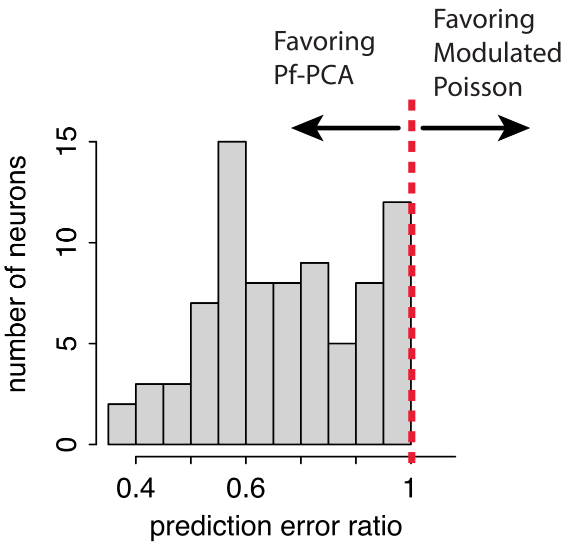

Supplementary Figure 1: Model assessment in the simulation study with bell-shape tuning curves (related to Fig. 2). Ratio of the prediction error of our Poisson fPCA model to that of the modulated Poisson model. Note that a ratio that is smaller than 1 means that Poisson fPCA model is favored.

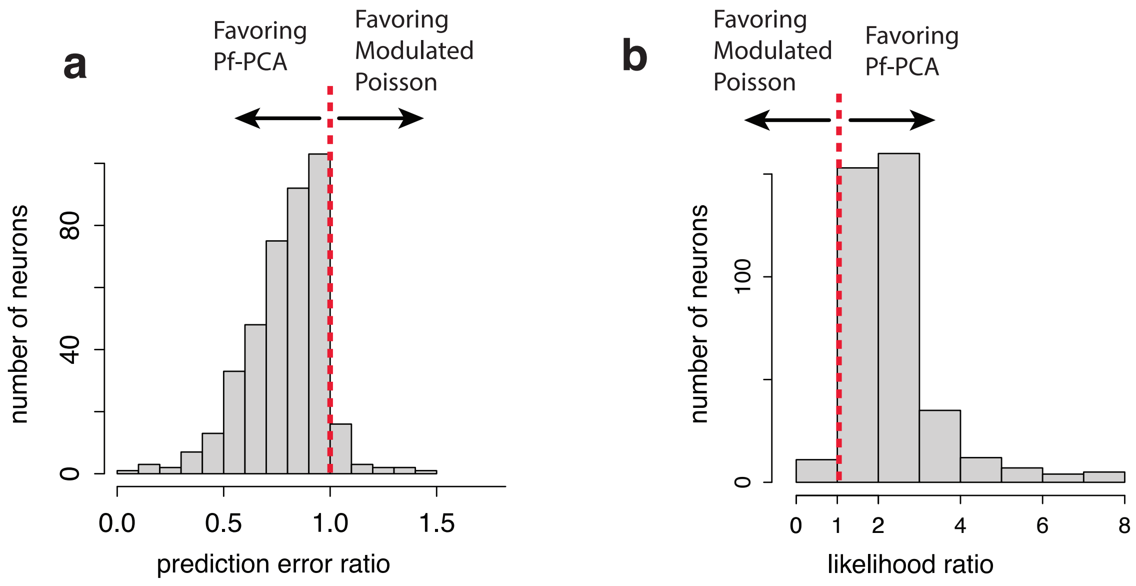

Supplementary Figure 2: Model assessment in the V1 datasets using cross-validation. (a) Ratio of the cross-validated prediction error of Poisson fPCA to that of modulated Poisson. (b) Ratio of the cross-validated likelihood of Poisson fPCA to that of modulated Poisson. According to both metrics, the Poisson fPCA model is favored over the modulated Poisson model.

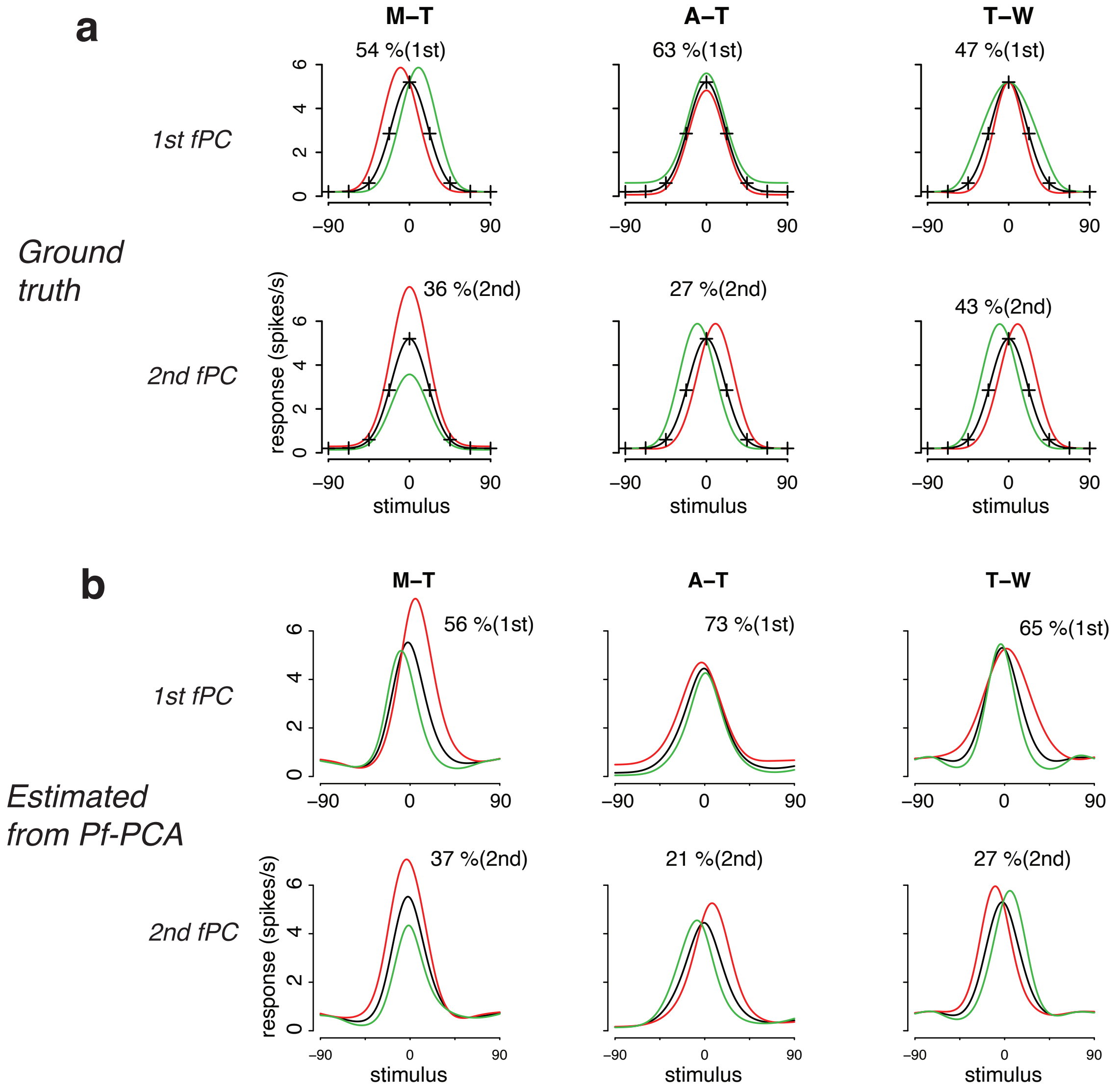

Supplementary Figure 3: Recovering mixed fluctuations with two components(related to Fig. 2). (a) Ground truth. M: multiplicative gain; T: tuning shift; W: tuning width change. M-T represents mixing multiplicative gain and tuning shift; same convention for the other two cases. Black curve: the tuning curve with zero fluctuation. This corresponds to the mean component exponentiated. Red/green curves: the corresponding tuning curves when setting the score to be ± 1 standard deviation of the scores. Crosses: the set of discrete stimuli used in the simulation. (b) Components recovered by Pf-PCA. The percentages show the proportions of variance explained by individual component. Color scheme: similar convention to (a).

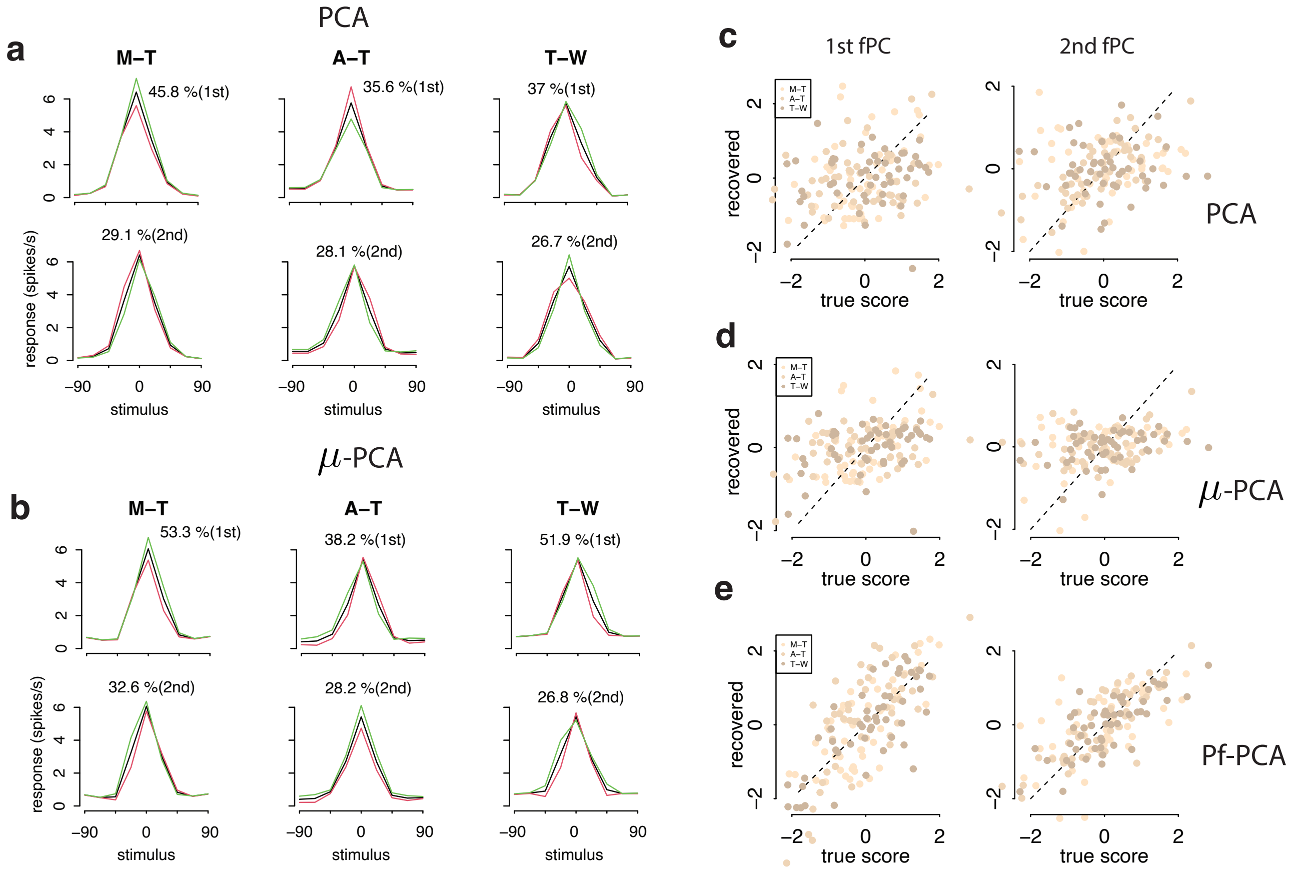

Supplementary Figure 4: Recovering mixed fluctuations with two components by PCA and μ-PCA (related to Supplementary Fig. 3). (a) Recovering mixed fluctuations with two components by PCA. (b) Recovering mixed fluctuations with two components by μ-PCA. Black curve: the tuning curve with zero fluctuation. This corresponds to the mean component exponentiated. Red/green curves: the corresponding tuning curves when setting the score to be ± 1 standard deviation of the estimated scores. (c,d,e). Recovering scores by PCA, μ-PCA, and Pf-PCA, respectively. In (a,b), the 1st row denotes the 1st component and the 2nd denotes the 2nd component. In (c,d,e), the 1st scatter denotes the 1st component and the 2nd denotes the 2nd component. In (c), the correlations are 0.22 and 0.31. In (d), the correlations are 0.35 and 0.27. In (e), the correlations are 0.76 and 0.72. M: multiplicative gain; T: tuning shift; W: tuning width change. M-T represents mixing multiplicative gain and tuning shift; same convention for the other two cases. The percentages show the proportions of variance explained by individual component.

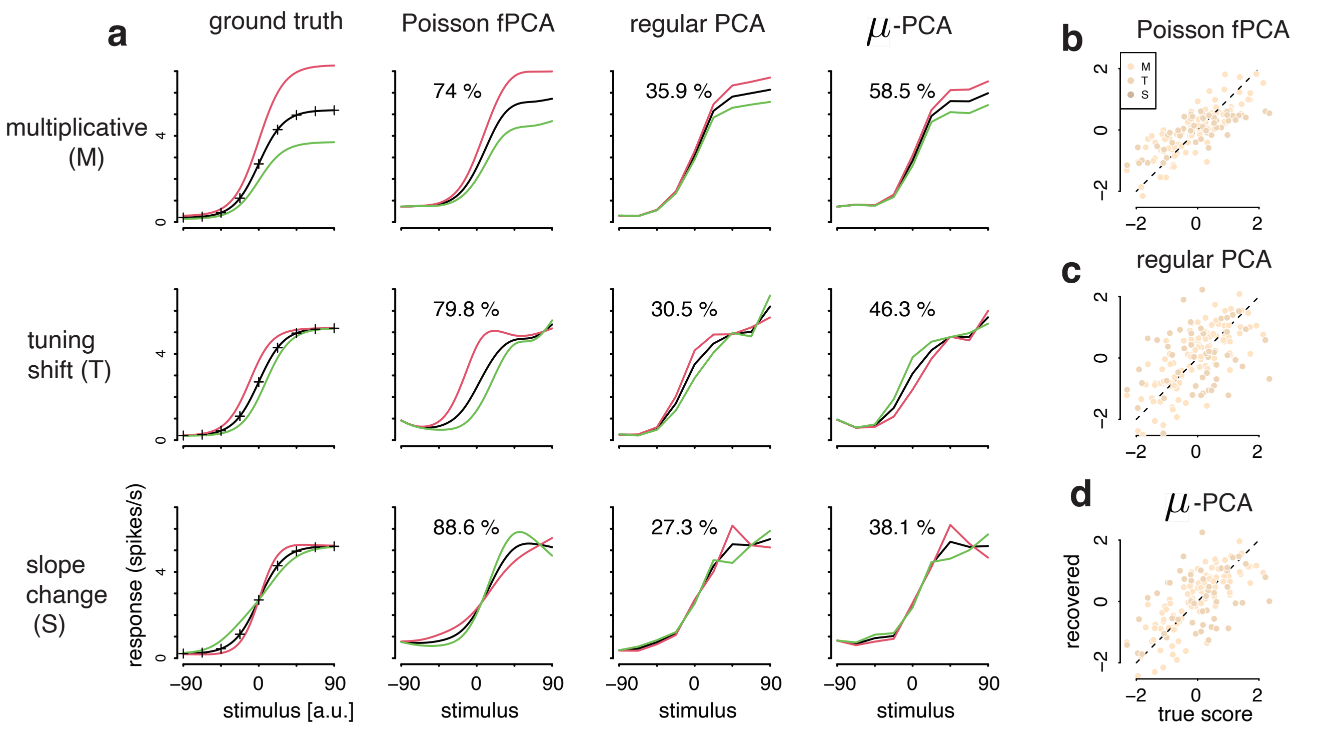

Supplementary Figure 5: Model validation with monotonic sigmoid response functions (related to Fig. 2 of the Main text). (a) Inferred fluctuations. Same convention as Fig. 2a. Note that in our simulation setting, the first fPC from “perfect" estimation procedure should explain 80% of the variance. (b,c,d) Recovering hidden scores for three methods. Light to dark bisque points denote three fluctuations cases: the multiplicative gain ("M"), the tuning shift ("T"), and the slope change ("S"). In (b,c,d), the correlations are 0.79, 0.43, and 0.45, respectively. Similar to the results for the bell-shape tuning curves, we found that our approach can recover the structure of fluctuations well. Thus our method is not limited by the specific shape of tuning curves.

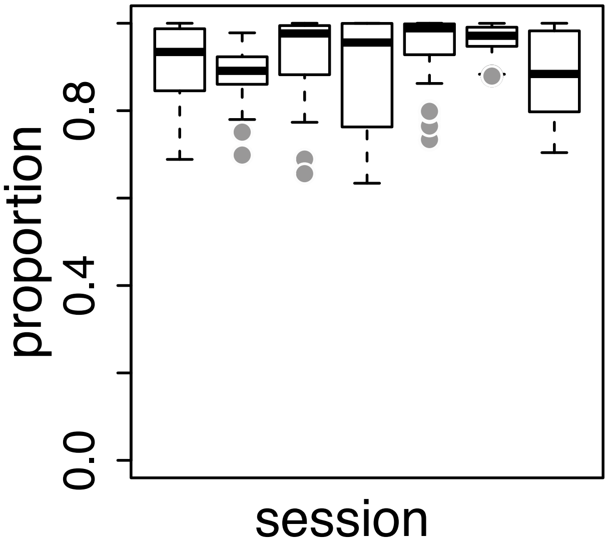

Supplementary Figure 6: The first three fPCs are sufficient for accounting most of the variance in the data. The average proportion of variance explained from D1 to D7 are 0.91, 0.89, 0.93, 0.90, 0.94, 0.96, and 0.89, respectively. Box plots: same convention as Fig. 3b.

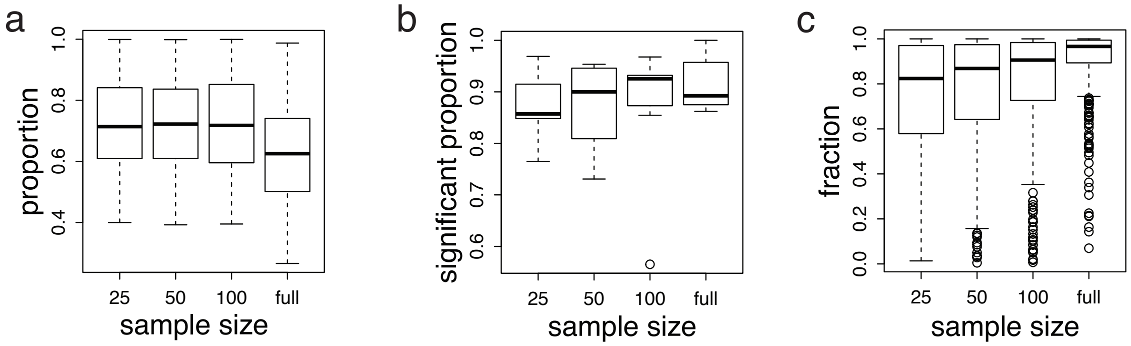

Supplementary Figure 7: Results of running Pf-PCA on V1 data with 25, 50, 100 blocks, and the full datasets. (a) Proportion of variance explained by the first fPC. (b) Proportion of neurons that show significant effect when regressing the first fPC against the mean component across seven datasets. (c) The “fraction" index when regressing the first fPC against the mean component using a linear model. Box plots: same convention as Fig. 3b.

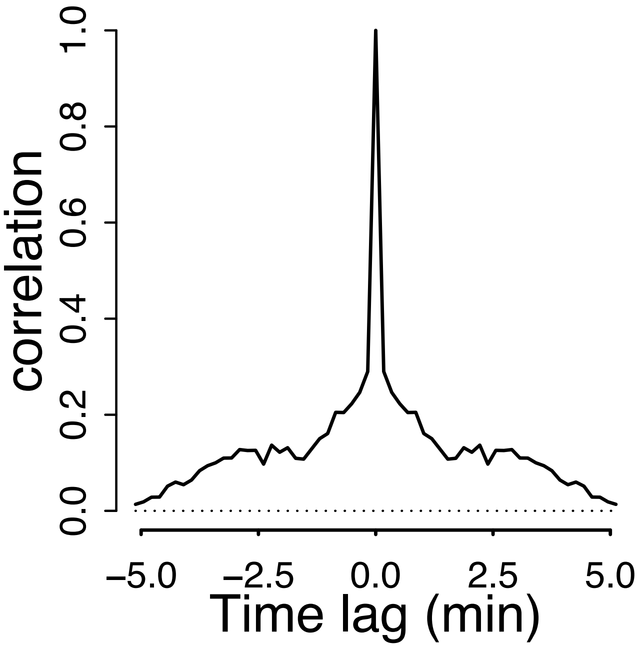

Supplementary Figure 8: The auto-correlation function of the estimated scores using the Pf-PCA method, averaged across all neurons from the seven datasets.

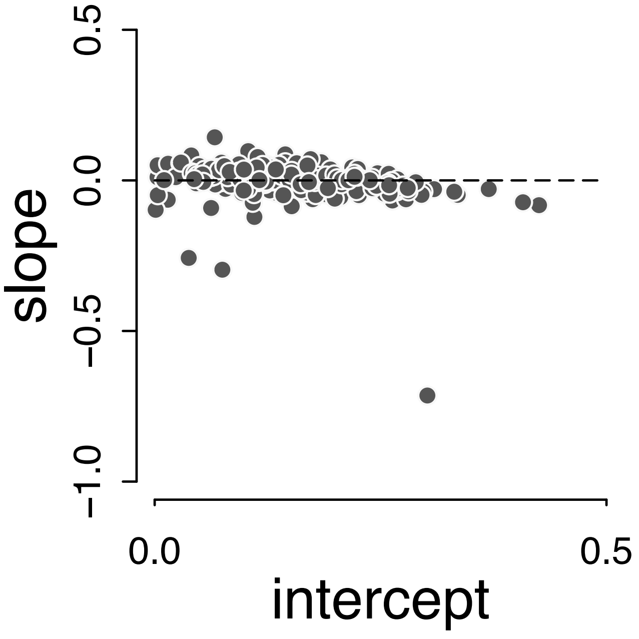

Supplementary Figure 9: Recovered slope and intercept from the synthetic datasets using Pf-PCA (related to Fig. 4). The synthetic datasets were generated assuming a pure multiplicative gain model while roughly matching individual neuron's firing rate in the real data. Two observations could be made: first, most of the neurons have a slope that is close to zero as expected; second, the recovered slope is flat over intercept.

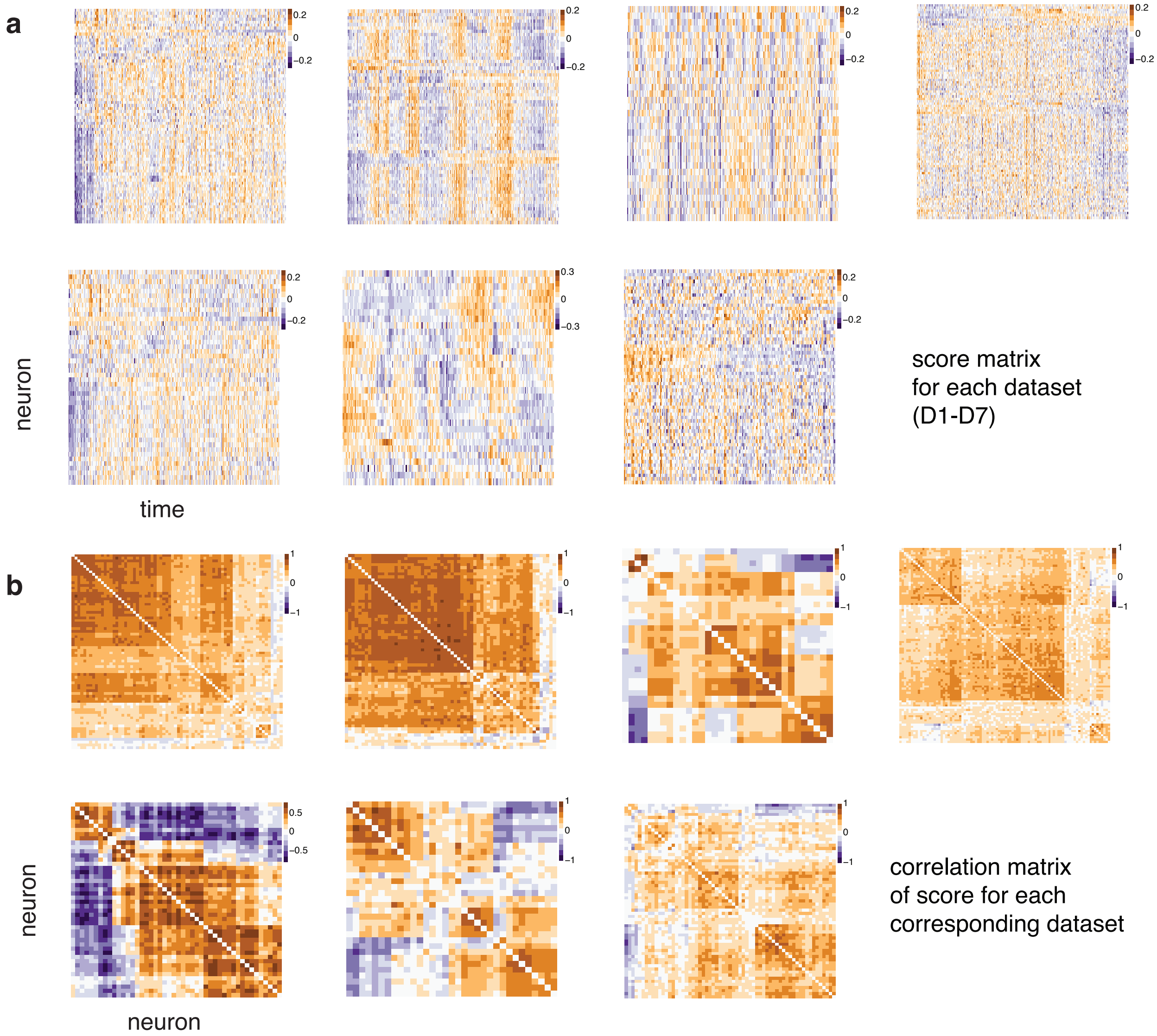

Supplementary Figure 10: Population structure of the tuning fluctuations in all datasets (related to Fig. 5). (a) The heatmap of scores for individual sessions (from D1 to D7). (b) The corresponding correlation matrix. Neurons were sorted using hierarchical clustering algorithm.

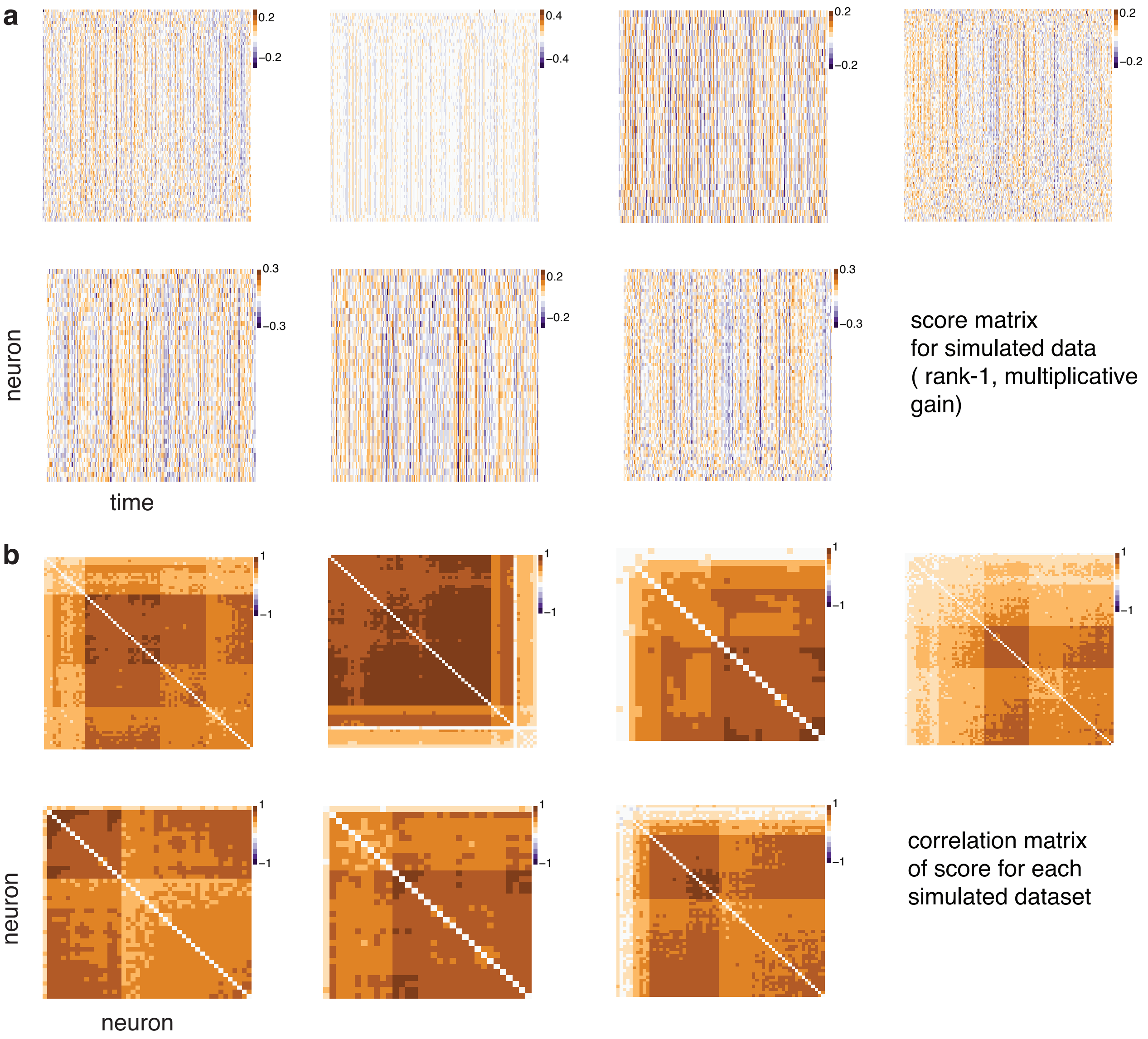

Supplementary Figure 11: Population structure of the tuning fluctuations in all simulated datasets based on multiplicative gain model (related to Fig. 5). (a)The heatmap of scores inferred from simulated datasets based on multiplicative gain model. (b) The corresponding correlation matrix. Neurons were sorted using hierarchical clustering algorithm.

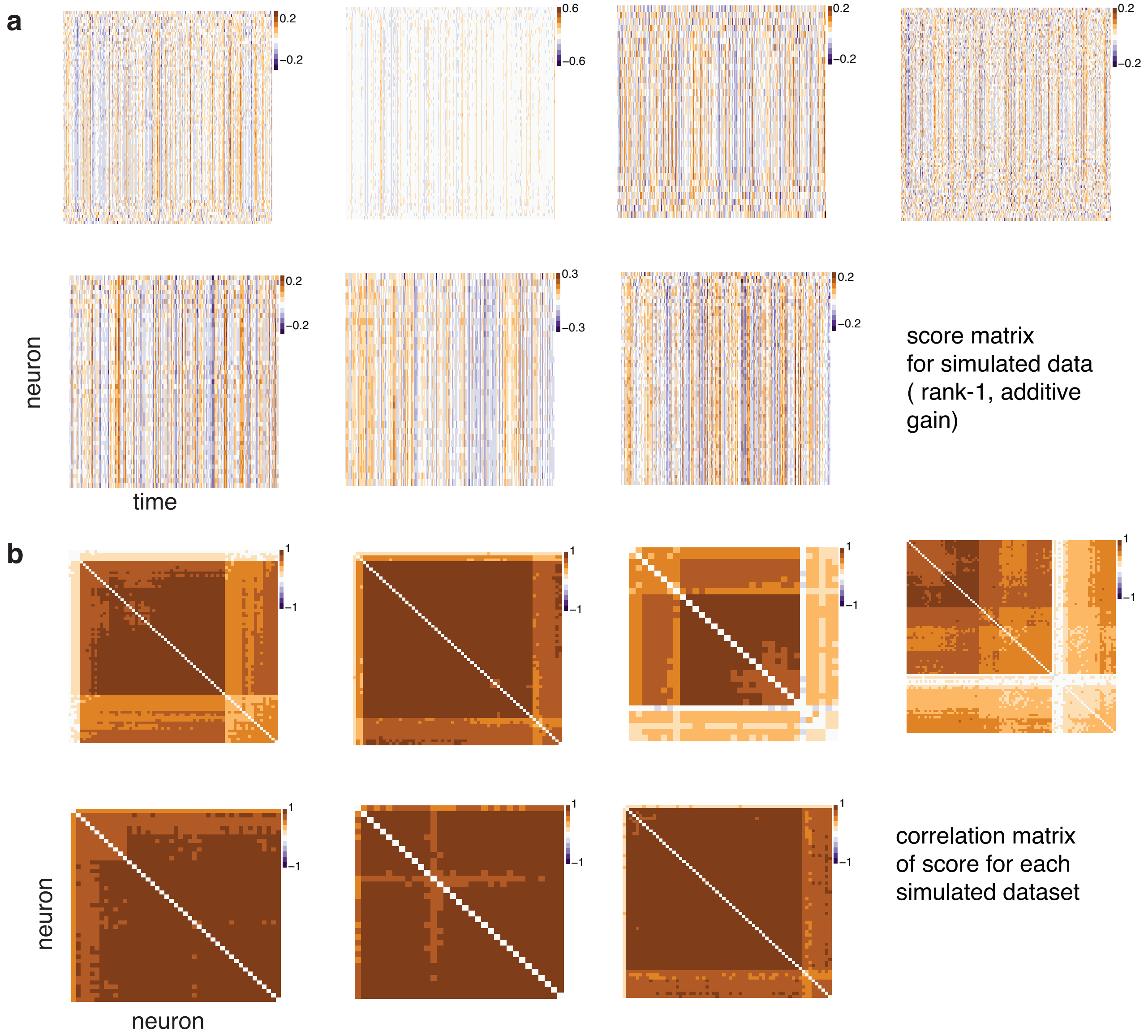

Supplementary Figure 12: Population structure of the tuning fluctuations in all simulated datasets based on additive model (related to Fig. 5). (a) The heatmap of scores inferred from simulated datasets based on additive model. (b) The corresponding correlation matrix. Neurons were sorted using hierarchical clustering algorithm.

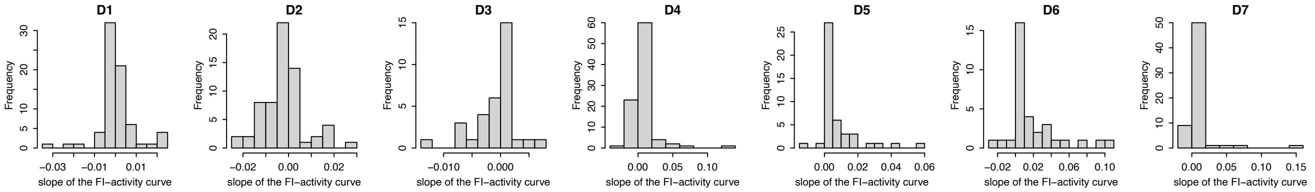

Supplementary Figure 13: Histogram of the slopes of the FI-activity curve for each session. The values of the slopes are generally centered around 0.

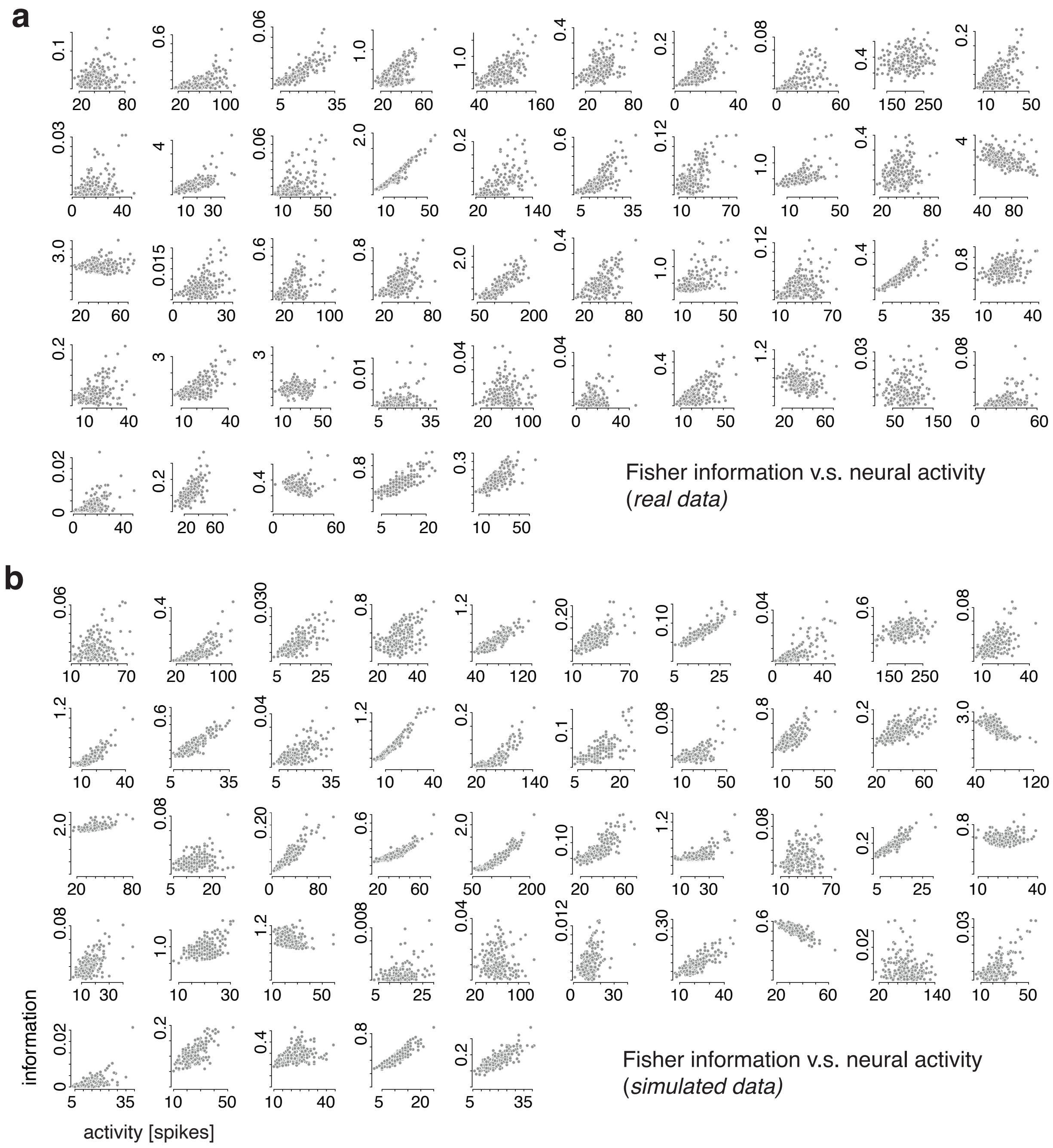

Supplementary Figure 14: Fisher information as a function of neural activity for individual neurons in one session of data (Session D5). (a) Fisher information as a function of the neural activity for each neuron. (b) Results from the recovery analysis. We simulated count data based on the estimated model by Pf-PCA, then ran our analysis pipeline to estimate the FI. The results suggest that our analysis pipeline is capable of recovering the relationship between the neural activity and the FI in the noise regime similar to the real data.

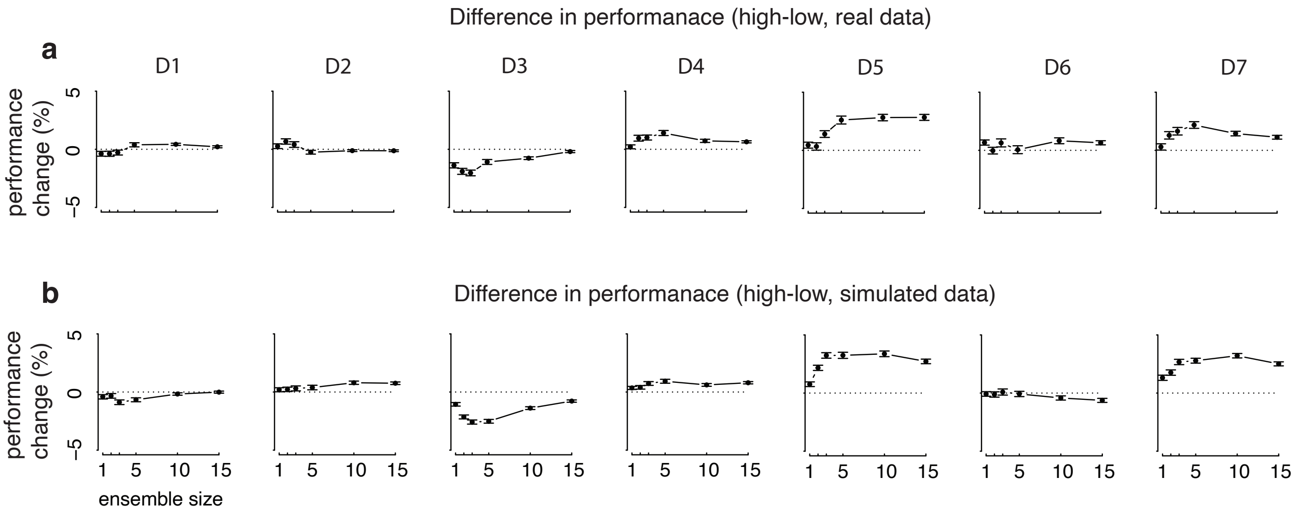

Supplementary Figure 15: Classification performance as a function of the number of neurons for both real data and simulated data. Error bar: standard deviation of the mean ($n = 500$ repeats). (a) performance change between high and low blocks (high minus low) in each dataset (D1- D7) based on classification procedure described in the Method section. (b) similar to panel (a), but for synthetic data. The results show that classification performance based on the synthetic data recapitulates the pattern in the real data. These observations together with the FI analysis, show that FI and classification performance based on splitting high/low blocks as done in [30] do not consistently map onto each other, suggesting the two analysis methods are characterizing non-identical information of the neural code. Note that datasets D1-D4 were from [30]. If one were to combine the performance across these four datasets, the results would show minimal difference between the low and high blocks (not shown), consistent with the results reported in [30].

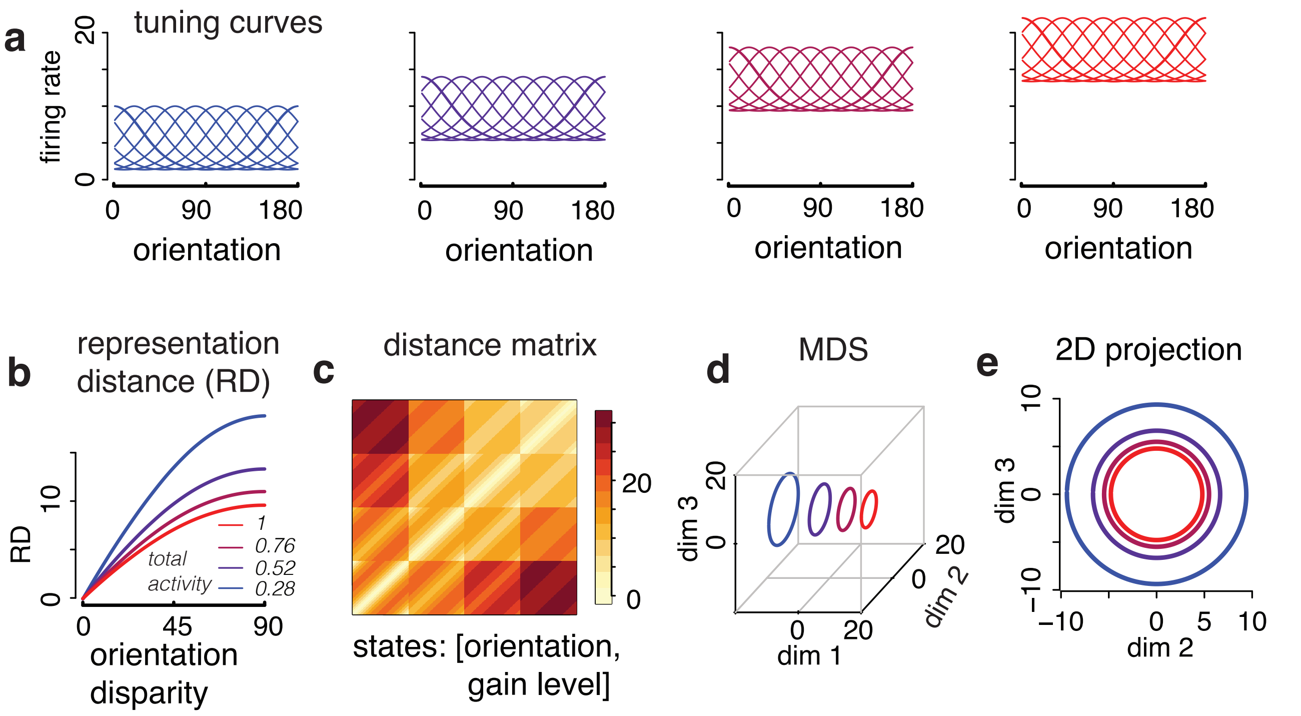

Supplementary Figure 16: Geometry analysis for additive model. (a-e) Similar convention to Fig. 7 (a-e), but for additive model. (a) Tuning curves for the population under four different levels of additive modulation. (b) The representation distance as a function of the orientation disparity. Inserted: the normalized total activity for each gain level. (c) The representational distance matrix for each pair of states, defined by the orientation and the gain level. We discretized the orientation into 180 bins, results in $180\times 4 = 720$ states. The states are arranged according to the orientation and the gain level. (d) Results from 3-D MDS. (e) Projection of the 3-D MDS results onto the second and third dimension reveals that the increased activity substantially reduced the size of the representation.
